# Prevalence of stimulant use and the role of opioid agonist treatment among people who inject drugs in France: Results from the COSINUS cohort study

**DOI:** 10.1111/dar.13955

**Published:** 2024-10-01

**Authors:** Perrine Roux, Aissatou Faye, Luis Sagaon‐Teyssier, Cécile Donadille, Laélia Briand Madrid, Maria Patrizia Carrieri, Gwenaelle Maradan, Marie Jauffret‐Roustide, Laurence Lalanne, Marc Auriacombe, the COSINUS study group

**Affiliations:** ^1^ Aix Marseille Univ, Inserm, IRD, SESSTIM, Sciences Economiques & Sociales de la Santé & Traitement de l'Information Médicale, ISSPAM Marseille France; ^2^ ORS PACA, Observatoire régional de la santé Provence‐Alpes‐Côte d'Azur Marseille France; ^3^ Centre d'étude des mouvements sociaux (Inserm U1276/UMR CNRS 8044/EHESS/Paris) Paris France; ^4^ British Columbia Center on Substance Use Vancouver Canada; ^5^ Baldy Center on Law and Social Policy, Buffalo University New York USA; ^6^ Institut Universitaire sur les Dépendances Montréal Canada; ^7^ INSERM 1329, Centre de recherche en biomédecine de Strasbourg Strasbourg Cedex France; ^8^ Department of Psychiatry and Addictology University Hospital of Strasbourg, Fédération de Médecine Translationnelle de Strasbourg Strasbourg France; ^9^ University of Bordeaux Bordeaux France; ^10^ Department of Psychiatry Perelman School of Medicine, University of Pennsylvania Philadelphia USA; ^11^ Addiction Team (Laboratoire de psychiatrie)/SANPSY CNRS USR 3413 Bordeaux France; ^12^ Pôle inter‐établissement Addictologie, CH Charles Perrens and CHU de Bordeaux Bordeaux France

**Keywords:** morphine, opioid agonist treatment, problematic use, stimulants, substitution

## Abstract

**Introduction:**

The co‐use of stimulants and opioids, including opioid agonist treatment (OAT), is very prevalent worldwide. A large body of data exists on the association between stimulant use and its health complications, and on OAT effectiveness among people with opioid use disorder. However, few data exist on stimulant‐opioid co‐use among people receiving OAT. Using data from the COSINUS cohort study, we investigated the association between the type of OAT and problematic stimulant use among persons who inject drugs (PWID).

**Methods:**

COSINUS is a 12‐month French cohort study of 665 PWID. Data were collected in face‐to‐face interviews at enrolment, at 6 and 12 months. We defined problematic stimulant use as daily use of and/or injecting stimulants. We used Bayesian model averaging (BMA) to identify factors associated with problematic stimulant use.

**Results:**

At baseline, 76% (*n* = 505) of the participants reported problematic stimulant use. The optimal model from the BMA estimation showed that, after adjusting on social precarity and daily injection, participants on prescribed morphine sulfate as an OAT (compared with methadone) and those who use daily unprescribed buprenorphine were less likely to report problematic stimulant use.

**Discussion and Conclusions:**

Our work highlights the high prevalence of problematic stimulant use among PWID in France but also the potential association between the type of OAT taken and stimulant use, by suggesting a protective effect of morphine sulfate on stimulant use. Since it has a higher intrinsic activity than other opioids, PWID on this OAT may be less interested in stimulants. Our findings warrant further investigation in clinical studies.

## 1 INTRODUCTION

The ever‐growing increase and diversification of stimulant use worldwide underlines the need for a greater understanding of the profiles and practices of people who use these drugs. A large body of literature has described the negative associations between stimulant use and health issues including its implication in overdoses [1], in cardiovascular diseases [2, 3], and infectious and psychosocial complications [4]. The US in particular is seeing a steep rise in mortality from stimulant use (mainly cocaine and methamphetamine), associated with greater availability of fentanyl [5]. The latter is used as an adulterant and is strongly associated with the third wave of the country's current opioid overdose crisis [5, 6]. The role of stimulant use in overdose mortality is widely described [1, 7]. Some authors have used the term ‘twin epidemics’ (i.e., overdose and stimulants/opioids) especially regarding increased use of methamphetamine among treatment‐seeking opioid users [8].

Although various psychosocial interventions have been implemented for stimulant use disorder [9], at the global level, no pharmacological intervention has yet been authorised to tackle it, despite extensive research programs on pharmacotherapies [10]. Some clinical trials have had promising results regarding the effectiveness of different candidate psychostimulant treatments for stimulant use disorder [11]. However, the effectiveness in these trials regarded very specific sub‐populations [12], such as people with attention‐deficit/hyperactivity disorder—a group which is particularly susceptible to stimulant use disorder—and people with no stimulant‐opioid co‐use disorder.

Stimulant use and stimulant use disorder are prevalent in very diverse sub‐populations of people who use drugs: people who use opioids [13], people with opioid use disorder (OUD) treated with opioid agonist therapy (OAT), people who use alcohol [14], people attending drug consumption rooms, homeless people [15]. As suggested by Brooks et al., all these sub‐populations have different rationales for using stimulants. Understanding these rationales is important to adapt harm reduction and treatment responses [16].

With regard to people with OUD treated with OAT, results from previous studies suggest that stimulant use in this sub‐population may be motivated by the fact that stimulants regulate or substitute the effect on opioids [8, 13]. Stimulant use has also been associated with greater difficulty in the management of OUD care in this sub‐population [17, 18]. For specifically, it has been linked to the continued use of unprescribed opioids [19, 20], patient‐perceived suboptimal OAT doses [21] and poorer treatment retention [22, 23]. These findings highlight the need to better understand how stakeholders experience stimulant use disorder, especially when it impacts OUD care.

Key findings were published in a recent qualitative study by Palis et al. [24] who documented how people that use stimulants self‐manage their use. Specifically, the authors found three interrelated categories of self‐management: distancing from the street environment, taking control of one's use, and seeking clinical and social support [24]. These findings suggest that a person's environment and available social support are key levers to help reduce problematic stimulant use in people who use drugs (PWUD).

The co‐use of stimulants and opioids is becoming increasingly prevalent [25], and the association between stimulant use and social precarity—including homelessness—is becoming ever stronger [26]. Moreover, several studies have shown that injection of cocaine by homeless people is linked to new HIV outbreaks [27, 28, 29].

In France, a national study by the French Addictovigilance Network underlined the high prevalence (32%) of cocaine injection among users who report cocaine‐related complications [30]. Moreover, the number of people who smoke crack and inject cocaine in the country is increasing, especially in those living in social precarity [31]. In contrast, methamphetamine consumption has not been observed in people with OUD living in precarity in France. Given that OUD is very prevalent in marginalised populations, and that no effective therapeutic response for problematic stimulant use currently exists, it is important to acquire a greater understanding of the potential correlates of this use, in order to adequately respond to this public health challenge. In this context, we used data from the COSINUS cohort study to identify factors associated with problematic stimulant use among PWID enrolled in harm reduction programs in France.

## 2 METHODS

### 2.1 Study design and population

COSINUS is a 12‐month multi‐site cohort study which aimed to evaluate the impact of drug consumption rooms on several health and socio‐behavioural outcomes in PWID. It was conducted between 2016 and 2018 in four French cities (Paris, Strasbourg, Bordeaux and Marseilles) among PWUD who met the following eligibility criteria: over 18 years old, could understand and speak French, and had injected either illegal drugs or a prescribed medication not destined for injection at least once in the previous month. Only the first two cities had a drug consumption room at the time of the study. Participants each had four face‐to‐face interviews with trained interviewers: at enrolment (M0), 3 months (M3), 6 months (M6) and 12 months (M12). More details can be found in the protocol article [32]. The study questionnaire collected information about socio‐demographic characteristics, substance use history, current substance use, health status and drug consumption room attendance. This study was approved by the Institutional Review Board (IRB00003888) of the French institute of medical research and health (opinion number: 14‐166) and by the National French Authority for Data Protection (approval number 915054‐06/05/2015).

### 2.2 Outcome and explanatory variables

Stimulant use was explored in all four COSINUS follow‐up questionnaires. However, data from the M3 questionnaire was not used in the present study, as we wished to look at regular timeframes, specifically 6‐month periods.

As we did not have a specific diagnosis tool to measure stimulant use disorder, we decided to use all the information declared by our participants in the questionnaire (frequency and route of administration) on the use of the following stimulants: cocaine, crack free base, speedball, amphetamines and unprescribed methylphenidate. We assumed that daily stimulant use or using stimulants by injection was a proxy of stimulant use disorder. We built a proxy outcome entitled ‘problematic stimulant use’, as follows:

- ‘yes’ if at least one of the listed stimulants was used every day (≥28 days a month) and/or mostly injected (i.e., as opposed to other administration routes);
- ‘no’ if not.

In addition to the items in this questionnaire section, we also used data from three questions in another section of the questionnaire which examined the use of prescribed stimulants. The first two questions were “Are you currently taking a prescribed medical treatment for another addiction [other than opioid addiction] (alcohol, cocaine, cannabis, etc.)?” and “Are you taking one or several prescribed medical treatments for other health issues?” Participants who answered yes to either question were asked to provide more details about their treatment in a dedicated space on the questionnaire. We verified all the responses relative to prescribed methylphenidate and its brand name Ritalin. The third question was “In the last month, have you ever injected your treatment?” Participants who indicated they injected their prescribed treatment were classified in the outcome's ‘yes’ category. Although this variable was not equivalent to using a diagnosis scale [33], we assumed that daily stimulant use or using stimulants by injection was a proxy of stimulant use disorder.

The following explanatory variables were considered as potential associated factors:

- Socio‐demographic and socio‐economic characteristics: city where interview took place, age, gender, education level (at least an upper‐secondary school certificate versus lower level), country of birth, living with a partner, type of housing (a) very stable (i.e., living in one's own house, in a rented home, or in family's home), (b) unstable (living in a hotel, at a friend's home), (c) very precarious (living in the street or a car/van or a squat) [34], employment status, receiving social welfare allowance, food aid (at least once in the previous month), health insurance, lifetime experience of prison, lifetime suicide attempt.
- Past and current drug use: time since first injection (<10 vs. ≥10 years) (M0); daily drug use during the previous month (heroin, unprescribed buprenorphine, unprescribed methadone, unprescribed morphine sulfate^1^ and cannabis), frequency of drug injection in the previous month (at least once daily vs. less often), harmful alcohol consumption (based on the Alcohol Use Disorders Identification Test‐C score of ≥3 for women and ≥4 for men).
- Prescribed treatments for opioid use disorder: (1) none, (2) methadone, (3) buprenorphine (or Subutex®, Suboxone®) and (4) morphine sulfate (or Skenan®) prescribed as an OAT.

### 2.3 Statistical analyses

We compared the baseline socio‐demographic, substance use, and health characteristics of participants with problematic stimulant use with those of participants without problematic stimulant use. Pearson's chi‐squared test (for categorical variables) and the Mann–Whitney *U* test (for continuous variables) were used to assess whether the differences between the two groups were significant or not. Our explanatory model aimed to study the association between problematic stimulant use and the type of OAT, by accounting for the model uncertainty created by the presence of several potential confounders. To achieve this, our estimations were performed as follows:

- Univariate analyses: To identify candidate factors associated with problematic stimulant consumption by implementing logistic regression.
- Logistic Bayesian model averaging (BMA): To account for model uncertainty [35, 36]. Instead of choosing a single ‘best’ model (e.g., using forward or backward procedures), BMA explores all possible model configurations. This has many benefits: first, it reduces overconfidence in a single model by accounting for and weighting the importance of different concurrent models; second, it produces consistent estimations in the presence of changing datasets (e.g., changes due to missing values in explanatory variables); third, it is robust when modelling misspecification due to omitted variables [35, 37].
  Moreover, for each covariate, the posterior probability (*p*! = 0) shows the probability that a variable will appear in the BMA model. In our context, this can be interpreted as the strength of the association of each covariate with problematic stimulant consumption.
  Covariates with a *p*‐value <0.25 were considered for the logistic BMA estimation.
- Mixed random‐effects model: To account for repeated measures (per individual) given the longitudinal nature of the dataset. The model identified from the BMA was used to specify an individual random effects model in order to obtain the final multivariable model.

All statistical analyses were performed using R version 4.1.2 (R: A language and environment for statistical computing. R Foundation for Statistical Computing, Vienna, Austria. URL https://www.R-project.org/) including the BMA package (version 3.18.17).

## 3 RESULTS

### 3.1 Description of the study sample

Table 1 describes the baseline characteristics of participants who had problematic stimulant use and those who did not. With regard to stimulant use at baseline, we found that 10.7%, 22.3% and 3.5% reported daily use of cocaine, crack and methylphenidate, respectively (only three and seven participants reported daily use of speedball and amphetamine, respectively). Three‐quarters (76%) of the sample reported stimulant use. The latter were more likely to have at least an upper‐secondary school diploma, live in very precarious housing, be unemployed and have no health insurance. They were also more likely to report daily cannabis use, but less likely to report unprescribed daily morphine sulfate use. Finally, they were more likely to declare taking methadone as an OAT, while non‐problematic stimulant users were more likely to take prescribed morphine sulphate as an OAT.

**TABLE 1:** Study population characteristics at enrolment according to problematic stimulant use or not (*n* [%] or median [IQR]); COSINUS study (*n* = 665).

	Problematic stimulant^g^ use			
	No	Yes	Total^f^	*p*
	*n* = 160 (24.1%)	*n* = 505 (75.9%)	665 (100.0%)	
City where study interview took place				0.912
Bordeaux	32 (20.0)	114 (22.6)	146 (22.0)	
Marseilles	50 (31.3)	149 (29.5)	199 (29.9)	
Paris	59 (36.9)	181 (35.8)	240 (36.1)	
Strasbourg	19 (11.9)	61 (12.1)	80 (12.0)	
Age, years, median (IQR)	39.5 (32–46)	37 (31–45)	38 (31–46)	0.387
Gender				0.126
Men	121 (75.6)	410 (81.2)	531 (79.8)	
Women	39 (24.4)	95 (18.8)	134 (20.2)	
Education level				0.007
Less than upper‐secondary school certificate	99 (61.9)	369 (73.1)	468 (70.4)	
At least upper‐secondary school certificate	61 (38.1)	136 (26.9)	197 (29.6)	
Country of birth				0.424
Born in France	129 (80.6)	421 (83.4)	550 (82.7)	
Born outside France	31 (19.4)	84 (16.6)	115 (17.3)	
Living with a partner^d^				0.320
No	115 (71.9)	382 (75.8)	497 (74.8)	
Yes	45 (28.1)	122 (24.2)	167 (25.2)	
Housing				<0.001
Very stable or stable	77 (48.1)	156 (30.9)	233 (35.0)	
Unstable	40 (25.0)	109 (21.6)	149 (22.4)	
Very precarious	43 (26.9)	240 (47.5)	283 (42.6)	
Employment (paid activity)				0.029
No	120 (75.0)	418 (82.8)	538 (80.9)	
Yes	40 (25.0)	87 (17.2)	127 (19.1)	
Receiving social welfare allowance				0.340
No	56 (35.0)	198 (39.2)	254 (38.2)	
Yes	104 (65.0)	307 (60.8)	411 (61.8)	
Received food aid at least once^a^				0.883
No	121 (75.6)	379 (75.0)	500 (75.2)	
Yes	39 (24.4)	126 (25.0)	165 (24.8)	
Health insurance				<0.001
No	22 (13.8)	150 (29.7)	172 (25.9)	
Yes	138 (86.3)	355 (70.3)	493 (74.1)	
Time since first drug injection, years				0.803
<10	53 (33.3)	162 (32.3)	215 (32.5)	
≥10	106 (66.7)	340 (67.7)	446 (67.5)	
Daily heroin use^a^, ^d^				0.527
No	154 (97.5)	487 (96.4)	641 (96.7)	
Yes	4 (2.5)	18 (3.6)	22 (3.3)	
Daily unprescribed buprenorphine use^a^, ^c^				0.733
No	123 (77.4)	384 (76.0)	507 (76.4)	
Yes	36 (22.6)	121 (24.0)	157 (23.6)	
Daily unprescribed methadone use^a^				0.105
No	108 (67.9)	307 (60.8)	415 (62.5)	
Yes	51 (32.1)	198 (39.2)	249 (37.5)	
Daily unprescribed morphine use^a^, ^c^				0.031
No	102 (64.2)	369 (73.1)	471 (70.9)	
Yes	57 (35.8)	136 (26.9)	193 (29.1)	
Daily cannabis use^a^, ^c^				0.021
No	116 (72.5)	315 (62.5)	431 (64.9)	
Yes	44 (27.5)	189 (37.5)	233 (35.1)	
Daily injection^a^, ^d^				0.887
No	66 (41.5)	206 (40.9)	272 (41.0)	
Yes	93 (58.5)	298 (59.1)	391 (59.0)	
Harmful alcohol consumption^b^				0.756
No	64 (40.0)	209 (41.4)	273 (41.1)	
Yes	96 (60.0)	296 (58.6)	392 (58.9)	
Opioid agonist treatment^c^				<0.001
None	55 (34.6)	189 (37.4)	244 (36.7)	
Methadone	49 (30.8)	185 (36.6)	234 (35.2)	
Buprenorphine	29 (18.2)	106 (21.0)	135 (20.3)	
Morphine sulfate	26 (16.4)	25 (5.0)	51 (7.7)	
Lifetime suicidal attempt^e^				0.26
No	104 (65.4)	299 (60.4)	403 (61.6)	
Yes	55 (34.6)	196 (39.6)	251 (38.4)	

^a^ In the previous month.

^b^ Alcohol Use Disorders Identification Test‐C score ≥3 for women and ≥4 for men.

^c^ 1 missing value.

^d^ 2 missing values.

^e^ 11 missing values.

^f^ For each variable the possible total was <665 if there were missing values (missing values not shown).

^g^ Stimulant = cocaine, crack or free base, speedball, amphetamines and unprescribed methylphenidate.

### 3.2 Factors associated with problematic stimulant use

The univariate model suggested 15 potential associated covariates (*p*‐value <0.25). All were considered in the BMA, implying the estimation of 2^15^ = 32,768 models accounting for all possible covariate combinations. The best 21 models suggested by the BMA accounted for 63% of the uncertainty of the model (cumulated posterior probability). The globally optimal model is presented in Table 2 and we show the best five models in Data S1, Supporting Information. According to the posterior probability, housing, daily injection and opioid agonist treatment were the most important covariates, as their probability of appearing in the estimated models was 100%. Socio‐economic factors including employment (*p* = 89.2%) and education level (*p* = 50.2%), as well as the use of daily unprescribed buprenorphine (*p* = 78.3%) were also important covariates, although their posterior probability was lower than 100%.

**TABLE 2:** Factors associated with problematic stimulant use. Univariate analysis using mixed‐logistic model and multivariable Bayesian Model Averaging and random‐effect mixed model; COSINUS study.

	Univariable analysis		Optimal model from BMA estimation		Mixed random‐effects model	
	*N* = 1466 visits. *n* = 665		*N* = 1455 visits. *n* = 664 (cumulative posterior probability 0.63)^c^, ^d^		*N* = 1455 visits. *n* = 664 (sigma = 2.4 95% CI [1.98. 2.91])^e^	
	OR [IC 95%]	*p*‐value	ORa [IC 95%]	*p*! = 0	ORa [IC 95%]	*p*‐value
Gender						
Men	Ref.					
Women	0.81 [0.43, 1.52]	0.505				
Age (continuous), years	0.99 [0.96, 1.02]	0.380				
Education level				50.2		
At least upper‐secondary school certificate	Ref.		Ref.		Ref.	
Less than upper‐secondary school certificate	2.63 [1.52, 4.57]	0.001	1.20 [0.80, 1.80]		2.30 [1.27, 4.14]	0.006
Country of birth				7.3		
France	Ref.		Ref.			
Outside France	0.50 [0.26, 0.95]	0.033	0.98 [0.83, 1.16]			
Living with a partner						
No	Ref.					
Yes	0.87 [0.52, 1.44]	0.576				
Housing				100		
Very stable or stable	Ref.		Ref.		Ref.	
Unstable	1.21 [0.72, 2.02]	0.474	1.10 [0.82, 1.49]		0.97 [0.56, 1.69]	0.909
Very precarious	4.54 [2.69, 7.65]	<0.001	2.17 [1.60, 2.94]		2.77 [1.56, 4.93]	0.001
Employment (paid activity)				89.2		
No	Ref.		Ref.		Ref.	
Yes	0.46 [0.28, 0.77]	0.003	0.63 [0.41, 0.97]		0.60 [0.34, 1.03]	0.066
Receiving social welfare allowance						
No	Ref					
Yes	0.72 [0.48, 1.09]	0.118				
Received food aid at least once^a^						
No	Ref.					
Yes	1.21 [0.78, 1.88]	0.390				
Health insurance				19.9		
No	Ref.				Ref	
Yes	0.30 [0.18, 0.51]	<0.001	0.93 [0.68, 1.28]		0.45 [0.25, 0.79]	0.005
Time since first injection, years						
<10	Ref.					
≥10	1.1 [0.65, 1.87]	0.727				
Daily heroin use^a^						
No	Ref.					
Yes	2.25 [0.56, 9.08]	0.254				
Daily unprescribed buprenorphine use^a^				78.3		
No	Ref.		Ref.		Ref.	
Yes	0.54 [0.24, 1.23]	0.143	0.54 [0.25, 1.17]		0.21 [0.09, 0.49]	<0.001
Daily unprescribed methadone use^a^				3.6		
No	Ref.		Ref.			
Yes	2.92 [0.65, 13.20]	0.163	1.02 [0.76, 1.38]			
Daily unprescribed morphine use^a^				3.7		
No	Ref.		Ref.			
Yes	1.56 [0.94, 2.59]	0.088	0.99 [0.87, 1.12]			
Daily cannabis use^a^				1.1		
No	Ref.		Ref.			
Yes	1.24 [0.81, 1.90]	0.330	1.00 [0.96, 1.04]			
Daily injection^a^				100		
No	Ref.		Ref.		Ref.	
Yes	2.56 [1.65, 3.98]	<0.001	1.65 [1.27, 2.15]		2.69 [1.67, 4.32]	<0.001
Harmful alcohol consumption^b^						
No	Ref.					
Yes	0.84 [0.55, 1.28]	0.409				
Opioid agonist treatment				100		
None	1.18 [0.70, 1.98]	0.536	0.96 [0.71, 1.30]		0.82 [0.47, 1.45]	0.501
Methadone	Ref.		Ref.		Ref.	
Buprenorphine	0.89 [0.48, 1.66]	0.723	0.99 [0.70, 1.30]		0.96 [0.48, 1.90]	0.903
Morphine sulfate	0.25 [0.12, 0.55]	0.001	0.32 [0.21, 0.50]		0.19 [0.08, 0.45]	<0.001
Monitoring				100		
M0	Ref.		Ref.		Ref.	
M6	0.48 [0.32, 0.70]	<0.001	0.65 [0.49, 0.87]		0.49 [0.33, 0.74]	0.001
M12	0.36 [0.24, 0.]	<0.001	0.53 [0.40, 0.71]		0.34 [0.23, 0.53]	<0.001

Abbreviations: CI, confidence interval; OR, odds ratio.

^a^ In the previous month.

^b^ Alcohol Use Disorders Identification Test‐C score ≥3 for women and ≥4 for men.

^c^ Indicates the part of the “uncertainty” accounted for by the 21 best models.

^d^ In BMA. *p*‐values are not reported as the final model is constructed using the weighted average of each effect (see Data S1). Instead. the *p*‐value “*p*! = 0” indicates the posterior probability of the variable appearing in the model (in percentage): that is, the probability that a given coefficient is not 0 (OR is not 1).

^e^ Corresponds to the variability accounted for by the individual random‐effects (i.e., control of the presence of repeated individual measures).

Moreover, the multivariate mixed random‐effects model indicated that participants living in a very precarious housing were more likely to report problematic stimulant use (adjusted odds‐ratio [95% confidence interval] = 2.77 [1.56, 4.93], *p*‐value = 0.001) compared to participants with stable or very stable housing, while the opposite was true for employed participants (0.60 [0.34, 1.03], *p*‐value = 0.066) compared with unemployed ones, and for those with health insurance (0.45, [0.25, 0.79], *p* = 0.005) compared with those without health insurance. With regard to the use of unprescribed psychoactive substances, participants who reported daily use of unprescribed buprenorphine were less likely to report problematic stimulant use, while no association was found with unprescribed methadone or morphine sulfate. Participants who declared daily injection (of any substance) were more likely to have problematic stimulant use (2.69 [1.67, 4.32], *p*‐value < 0.001). Finally, with regard to prescribed opioids, participants taking morphine sulfate as an OAT were less likely to report problematic stimulant use (0.19 [0.08, 0.45], *p*‐value < 0.001) compared with those taking methadone as an OAT.

Controlling for the follow‐up time point (i.e., M0, M6 and M12) ensured that attrition did not modify the estimations. Participants who were still in the study at M6 and M12 were less likely to report problematic stimulant use (i.e., study outcome) at M6 (0.49 [0.33, 0.74]) and at M12 (0.34 [0.23, 0.53]).

## 4 DISCUSSION

The main findings of this longitudinal study are the high prevalence of stimulant use among PWID in the cohort and the positive association between the prescription of morphine sulfate as an OAT on problematic stimulant use among people with opioid use disorder. While these two results need to be contextualised, as we had a relatively small sample and a short follow‐up period, they highlight an interesting association between the type of OAT taken by PWID enrolled in harm reduction programs and concurrent stimulant use. More specifically, they show that participants who were prescribed morphine sulfate as an OAT (compared to those prescribed methadone) were less likely to have problematic stimulant use while no difference was observed between buprenorphine and methadone as an OAT. PWID using daily unprescribed buprenorphine were also less likely to report problematic stimulant use.

For people with OUD, OAT initiation can be complex and it takes time to achieve a level of stability. Polydrug use, specifically simultaneous stimulant use while on OAT treatment, has been studied without consistent findings. Very few studies to date have focused on the role which the type of OAT taken plays in stimulant‐opioid co‐use.

Our findings show interesting associations between the type of opioid used and problematic stimulant use, reflecting results from previous studies. The first is a previous study by our team, where we investigated 240 PWID enrolled in harm reduction programs; those who reported using morphine sulfate were less likely to use stimulants [38]. The other study was conducted in a hospital setting in Zurich among 105 patients on OAT. There, the authors found that persons treated with methadone were more likely to have a positive hair test for cocaine than patients treated with buprenorphine [39]. Although we found no difference between prescribed methadone and prescribed buprenorphine in our present study, people with unprescribed daily buprenorphine use were less likely to report problematic stimulant use. Unprescribed buprenorphine has been widely accessible on the black market in France since the early 1990s [40], especially through doctor shopping practices [41]. Our result for unprescribed buprenorphine needs more detailed investigation to understand the causal direction of the association.

We found that receiving prescribed morphine sulfate as an OAT may be associated with a lower likelihood of problematic stimulant use. One possible explanation for this is that morphine sulfate may be a more effective substitute treatment for people with OUD who do not simultaneously take stimulants. Another is that access to this OAT is more difficult, possibly because morphine sulfate is prescribed off‐label (for OUD) and tends to be given to more medically and socioeconomically stabilised patients. However, our statistical model took both of these factors (i.e., unprescribed opioids and socio‐economic characteristics) into account. A previous French article found that while morphine sulfate was not commonly prescribed as an OAT for OUD, 14% of PWID declared using it in the previous month [42]. Another study found that a non‐negligible proportion of people with OUD had requested it from health providers [43]. Specifically, the latter study showed that the prevalence of morphine sulfate use in 19 harm reduction/addiction services was 7% in 2012. In the same article, the authors presented the findings of another study, where data collected from a self‐administered questionnaire among 83 morphine sulfate users suggested that its use can be explained by the fact that users perceive it to be similar to heroin. They also reported that the main route of administration was intravenous injection, despite complications associated with this route [44]. All these findings suggest that involving the patient in the decision on which OAT to take, and investigating what effect the patient would like to experience from an OAT, should be two central elements of OUD management [45]. It is important to note that morphine sulfate is prescribed off‐label as an OAT for OUD in France, and is known to be associated with risk of overdoses and complications [44]. To fulfil the wishes of the sizeable proportion of PWID who would like to receive injectable morphine sulfate as an OAT [46], it is essential to organise a safe and well organised OAT distribution system.

An ever‐growing body of literature is investigating the consequences of stimulant‐opioid co‐use on public health, especially in the US context where some authors refer to ‘twin epidemics’ [8, 47]. However, few qualitative studies have tried to disentangle the reasons and motivations associated with this co‐use. Some studies highlighted that the co‐use of psychoactive substances is motivated by the desired effect of the drug combination, whether that be a specific ‘combined’ effect (sedation, euphoria, etc.), or using one drug to attenuate the effects of the other [48, 49]. Our findings suggest that morphine sulfate could be a better OAT option for some patients with OUD, because of its higher intrinsic activity than other OAT [50, 51]; however, this could only take place in a context where the risk of overdoses and other complications are prevented through adapted and well‐regulated distribution. Specifically, it may prevent them from co‐using unprescribed stimulants to compensate for what they consider to be a sub‐optimal effect of their opioid‐based OAT. Nevertheless, public health responses, including medical treatment, do not often consider this synergistic drug effect among people practicing polydrug use. This was highlighted in another qualitative study which showed that PWUD described the co‐use of opioids and methamphetamine as a harm reduction strategy for many reasons (managing their highs, balancing the effects of each drug, and managing opioid withdrawal symptoms). Moreover, that article underlined that co‐users are influenced by interrelated structural, community and individual‐level factors [52]. This underlines the importance of understanding motivations and expectations regarding co‐use from the PWUD perspective.

We found several other correlates of problematic stimulant use which were previously published, but which are nonetheless important to mention here. Specifically, a lower educational level, unemployment, no health insurance, and very precarious housing were all associated with a higher likelihood of problematic stimulant use (reflecting previous findings [53]). This corroborates previous results showing that among people experiencing structural vulnerabilities, especially homeless persons, stimulant‐opioid co‐use is prevalent [54]. It is important to take these vulnerabilities into account in clinical research and harm reduction interventions, especially since various clinical studies often exclude people facing structural vulnerabilities [55, 56] who are ‘hard to reach and retain’ [57, 58]. Such an approach has been promoted in Rhodes ‘risk environment framework’ which asserts that people who use drugs face complicated situations because of their difficult living and social conditions [59]. In this context, a new framework where housing interventions are part of the response to these populations is needed [57, 60] to tackle complications associated with stimulant use among people with very precarious housing.

In terms of clinical perspectives, the link between the type of OAT and stimulant use which we observed needs to be investigated in greater detail. This would help care providers to choose the most suitable type of OAT together with the patient, as part of a patient‐centred approach. In addition, our results advocate adequate responses to problematic stimulant use. One response could be to continue promoting research to identify effective stimulant agonist treatment [11]. Some interesting avenues for research, especially with dexamphetamine, has been suggested in clinical trials targeting patients medically treated with heroin who have stimulant use disorder [61]. Existing data advocate a new treatment paradigm which promotes medical and psychosocial interventions, social support mechanisms, and harm reduction interventions to adequately answer the problem of stimulant use disorder [62]. Moreover, the contingency management approach was presented as the most promising psychosocial intervention in a recent Cochrane report [9]. Another response could be to experiment with safe supply needs; this intervention is already being implemented in the context of the current worldwide opioid overdose crisis, where stimulants are adulterated with potent opioids [63]. Furthermore, experimental work on access to off‐label psychostimulant prescription is ongoing [64].

Some study limitations must be acknowledged. First, the data used were self‐reported which may have led to desirability bias. However, the data were collected by trained interviewers and self‐reports have been already recognised as valid [65]. Second, the population of PWID in France is heterogeneous (i.e., desired effects, metabolism, etc.) and the level of opioid dependence may differ from one profile to another. Third, we had no data on mental health diagnosis in our questionnaire, except for a history of suicide attempts (which were not associated with the outcome). This is unfortunate given that problematic stimulant use disorder is associated with psychiatric comorbidities. The impact of psychiatric comorbidities on problematic stimulant use should be investigated in greater detail in future prospective research. Fourth, our data were collected in 2018. Fifth, the small sample sizes, especially in the morphine sulfate group (due to reduced access to this OAT), limit study power. Finally, participants who were more likely to drop out reported more problematic stimulant use; this may have biased our findings. It also suggests that a different methodology is needed to better investigate this sub‐population in the future.

In conclusion, our findings provide indications as to how we can better address problematic stimulant use in populations with accumulated vulnerabilities such as social precarity and OUD. Existing harm reduction services and access to OAT must be adapted for people with problematic stimulant use. To achieve this, an improved patient‐centred approach is required which ensures patients receive adequate treatment.

## AUTHOR CONTRIBUTIONS

Study conception and design: MPC, LL, MA, MJR, PR. LST and AF performed the statistical analyses. PR and MA drafted the first manuscript. MA, MJR, LL, AF, LST, MPC and LBM revised the manuscript. GM supervised the study‐site interviewers and contributed to improving the design of the study. The Cosinus study group contributed to the study implementation. All authors substantially contributed to the manuscript and approved the final version.

## CONFLICT OF INTEREST STATEMENT

The authors declare that they have nothing to disclose regarding funding or conflict of interest with respect to this manuscript.

## Supporting information


**Table S1.** Bayesian model averaging estimation. Five best models.

## Endnote

^1^
The use of other prescription opioids is quasi inexistent among PWID; accordingly, we excluded this modality from the analyses.

## 1

Members of the COSINUS study group:

Marc Auriacombe; Cyril Berenger; Gilles Bertoia; Laélia Briand Madrid; Maria Patrizia Carrieri; Isabelle Célérier; Carole Chauvin; Manon Chevalier; Jean‐Marie Danion; Sébastien de Dinechin; Cécile Denis; Natascia Grelli; Marie Gutowski; Naomi Hamelin; Marie Jauffret‐Roustide; Charlotte Kervran; Sébastien Kirchherr; Laurence Lalanne; Mireille Le Breton; Gwenaëlle Maradan; Sarah Moriceau; Perrine Roux; Antoine Vilotitch.
