# Supplementary material for: Prevalence of stimulant use and the role of opioid agonist treatment among people who inject drugs in France: Results from the COSINUS cohort study
Source: Drug Alcohol Rev. 2024 Oct 1;44(1):275–87. doi: 10.1111/dar.13955 (PMC11743017; doi:10.1111/dar.13955)
Supplement: Supplementary file 1 — Table S1. Bayesian model averaging estimation. Five best models. [file DAR-44-275-s001.docx]

**Table S1. Bayesian model averaging** **estimation. Five best models**

| **Variables** | **p!=0** | **EV** | **SD** | **Model 1** | **Model 2** | **Model 3** | **Model 4** | **Model 5** |
| --- | --- | --- | --- | --- | --- | --- | --- | --- |
| Intercept | 100 | 0.7833 | 0.263 | 0.62 | 0.85 | 0.86 | 1.2 | 0.94 |
| Gender | 0.0 |  |  |  |  |  |  |  |
| Women | 0.0 | 0 | 0 |  |  |  |  |  |
| Age (continuous), years |  | 0 | 0 |  |  |  |  |  |
| Education level | **50.2** |  |  |  |  |  |  |  |
| Less than upper-secondary school certificate |  | 0.1837 | 0.206 | 0.36 |  |  |  | 0.36 |
| Country of birth | **7.3** |  |  |  |  |  |  |  |
| Outside France |  | -0.0203 | 0.084 |  |  |  |  |  |
| Living with a partner | 0.0 |  |  |  |  |  |  |  |
| Yes |  | 0 | 0 |  |  |  |  |  |
| Housing | **100.0** |  |  |  |  |  |  |  |
| Unstable |  | 0.0996 | 0.151 | 0.079 | 0.11 | 0.12 | 0.089 | 0.056 |
| Very precarious |  | 0.7736 | 0.155 | 0.76 | 0.8 | 0.79 | 0.73 | 0.69 |
| Employment (paid activity) | **89.2** |  |  |  |  |  |  |  |
| Yes |  | -0.4664 | 0.22 | -0.49 | -0.56 | -0.55 | -0.54 | -0.47 |
| Health insurance | **19.9** |  |  |  |  |  |  |  |
| Yes |  | -0.0721 | 0.163 |  |  |  | -0.36 | -0.36 |
| Daily unprescribed buprenorphine use | **78.3** |  |  |  |  |  |  |  |
| Yes |  | -0.6179 | 0.394 | -0.8 | -0.77 |  | -0.78 | -0.81 |
| Daily unprescribed methadone use^a^ | **3.6** |  |  |  |  |  |  |  |
| Yes |  | 0.024 | 0.151 |  |  |  |  |  |
| Daily unprescribed morphine use^a^ | **3.7** |  |  |  |  |  |  |  |
| Yes |  | -0.0104 | 0.063 |  |  |  |  |  |
| Daily cannabis use^a^ | **1.1** |  |  |  |  |  |  |  |
| Yes |  | 0.0017 | 0.021 |  |  |  |  |  |
| Daily injection^a^ | **100.0** |  |  |  |  |  |  |  |
| Yes |  | 0.502 | 0.135 | 0.52 | 0.51 | 0.45 | 0.5 | 0.51 |
| Opioid agonist treatment | **100.0** |  |  |  |  |  |  |  |
| No |  | -0.0367 | 0.154 | -0.0059 | -0.023 | -0.083 | -0.071 | -0.053 |
| Buprenorphine |  | -0.0063 | 0.179 | 0.003 | 0.023 | -0.097 | 0.055 | 0.036 |
| Morphine sulfate |  | -1.1298 | 0.219 | -1.1 | -1.1 | -1.1 | -1.1 | -1.1 |
| Monitoring | **100.0** |  |  |  |  |  |  |  |
| 6 months |  | -0.4327 | 0.147 | -0.44 | -0.44 | -0.42 | -0.44 | -0.44 |
| 12 months |  | -0.6338 | 0.148 | -0.65 | -0.64 | -0.6 | -0.64 | -0.64 |
| Number of variables in the model |  |  |  | 7 | 6 | 5 | 7 | 8 |
| Bayesian information criterion) |  |  |  | -8800 | -8800 | -8800 | -8800 | -8800 |
| Posterior probability of the model |  |  |  | 0.213 | 0.213 | 0.086 | 0.062 | 0.061 |

p!=0: the posterior probability that each variable is non-zero (percentage). EV: posterior coefficient expected value. SD: the posterior standard deviation of each coefficient (from model averaging).
